# Colleague relationships as a stress factor for special needs school teachers: A comparison with other public schools

**DOI:** 10.1002/pcn5.70061

**Published:** 2025-02-03

**Authors:** Masateru Matsushita, Schuhei Yamamura

**Affiliations:** ^1^ Department of Psychology, Faculty of Human Sciences Konan Women's University Kobe Japan; ^2^ Department of Psychiatry Kinki Central Hospital of the Mutual Aid Association of Public School Teachers Itami Japan

**Keywords:** conflict with co‐workers, job satisfaction, peer relationships, special needs school, stress

## Abstract

**Aim:**

Although teachers in special needs schools do not always work long hours, their sick leave rate is higher than that of teachers in other types of schools. We aimed to compare the causes of stress between teachers in public special needs schools and different public schools and explore the stress factors among teachers in special needs schools.

**Methods:**

An Internet‐based cross‐sectional survey was conducted between June and December 2021. We analyzed the data of 203,433 teachers with no missing values, including 15,159 teachers from special needs schools. Demographic variables, stress factors, psychological and physical stress responses, job satisfaction, and working hours were analyzed.

**Results:**

Among high‐stress teachers, 30.4% of the special needs school teachers had difficulties with their relationships with their colleagues; these difficulties were statistically significantly more frequent than for teachers in other school types (15.1% in elementary, 17.6% in junior high, and 19.1% in high schools). Multiple logistic regression analysis revealed that high‐stress teachers in special needs schools were 1.82 times more likely than teachers in other types of schools to be stressed by their relationships with colleagues after controlling for age, sex, teaching experience, size of school, working hours per day, and job satisfaction (95% confidential interval = 1.65–2.01, Wald = 137.3, *p* ≤ 0.001).

**Conclusion:**

The findings suggest that not only a reduction in working hours but also the cultivation of human relationships and a cooperative working environment in the workplace may be effective in eliminating stress among teachers in special needs schools.

## INTRODUCTION

In 2023, 98.7% of elementary school students, 92.2% of junior high school students, 65.3% of high school students, and 59.3% of special needs students participated in public schools.^1^ Public schools play a dominant role in Japanese education. Conversely, the Ministry of Education, Culture, Sports, Science, and Technology (MEXT) has revealed that the number of teachers in public schools on leave due to mental illness reached a record high of 6539 in 2022.^2^ Some concerns about taking sick leave will lead to subsequent resignations and a more apparent shortage of teachers, which will adversely affect student learning. The chronic leave for teachers responsible for public education is a critical concern in the Japanese education system.

One reason for teachers' leave due to mental illness could be long overtime working hours.^3^ The long working hours of Japanese teachers are widely recognized. In 2018, the Teaching and Learning International Survey (TALIS) by the Organization for Economic Cooperation and Development (OECD) examined the working environments of secondary school teachers across 48 countries and territories. The survey reported that Japanese teachers worked 56 h per week, the most extended of any country surveyed.^4^ Previous studies have shown that long working hours are significantly associated with psychological distress and symptoms of depression or insomnia and affect teachers' mental health.^5^, ^6^, ^7^, ^8^, ^9^ Therefore, the MEXT is actively discussing a policy to improve long working hours and reform the working environment for teachers in public schools.

While teachers in special needs schools may not always work overtime, some complain of mental and physical illnesses.^10^, ^11^, ^12^ The percentage of teachers working more than 80 h of extra overtime per month, considered the benchmark for sudden death from overwork in Japan, was 4.4% for elementary school teachers, 13.7% for junior high school teachers, and 10.3% for high school teachers. The percentage of teachers working extreme overtime was 1.8% for teachers in special needs schools, a relatively minor percentage.^13^ However, the rate of teachers on leave due to mental illness in 2021 was highest in special needs schools at 0.85%, compared to 0.71% in elementary schools, 0.61% in middle schools, and 0.42% in high schools.^14^ These findings suggest that stress factors besides working hours may influence physical and mental health problems among teachers in special needs schools, resulting in lengthy absences from work. Given the high number of teachers on leave in special needs schools, it is essential to identify what the high‐stress teachers in special schools perceive as burdensome about their work.

However, job satisfaction can function as a protective factor for mental health. It has been reported that high job satisfaction is associated with favorable mental health among teachers in Japan, as well as in other countries.^15^, ^16^, ^17^, ^18^, ^19^, ^20^ Given that job satisfaction may be a buffer against stressors among teachers in special needs schools, it is important to adjust for the effect of job satisfaction as a confounding factor.

This study aimed to compare stressors among teachers in special needs schools and other public schools and to identify stressors among high‐stress teachers in special needs schools. Furthermore, after adjusting for working hours and job satisfaction, we compared the stressors identified between teachers in special needs schools and those in other schools.

## METHODS

### Participants and procedure

We used data from the Stress Check Program conducted nationwide between June and December 2021 by the Public‐School Teachers' Mutual Aid Association for Public School Employees. The survey was conducted using a web‐based questionnaire related to the participants' characteristics, occupational stress, and self‐evaluated working hours per day. A total of 323,584 teachers and administrators from special needs schools, elementary, junior high, and high schools, and public universities responded to the survey. Of the 255,263 teachers across special needs, elementary, junior high, and high schools, 203,433 tenured teachers with complete responses were included in the analysis. This group included: (1) 15,159 special needs school teachers (mean age 44.1 ± 10.94 years; 9,327 or 61.5% women), (2) 95,991 elementary school teachers (mean age 41.6 ± 12.19 years; 59,445 or 61.9% women), (3) 54,684 junior high school teachers (mean age 42.0 ± 11.93 years; 23,165 or 42.4% women), and (4) 37,599 high school teachers (mean age 46.0 ± 10.98 years; 12,539 or 33.3% women).

Teachers in various positions and employment statuses staff public schools in Japan. For example, there are principals and vice principals in administrative positions, tenured teachers with no fixed terms of employment, and fixed‐term teachers whose contracts must be renewed every year. Their duties vary significantly depending on their position and employment status. Therefore, the most common type of teacher, the tenured teacher, was the subject of this study.

Information on sex, age, number of years of continuous employment at the current school, number of faculty members at the current school, hours worked per day, occupational stress, job satisfaction, and type of current school were analyzed.

### Measurement

The Brief Job Stress Questionnaire (BJSQ) was used to assess whether teachers experienced high levels of occupational stress. The BJSQ is widely used in occupational health in Japan and is an established questionnaire for helping workers identify their stress conditions and detect problems at an early stage.^21^, ^22^, ^23^ It is a 57‐item scale designed to assess the following three aspects of work: psychological job demands and job control (17 items); psychological and physical stress responses (29 items); and buffering factors, such as social support at the workplace (11 items). The BJSQ has been shown to have adequate reliability, consistency, and validity.^24^

In this study, the total score of psychological and physical stress responses was used to indicate high‐stress workers. Each item is rated on a four‐point Likert like scale (1 = *almost never*, 2 = *sometimes*, 3 = *often*, 4 = *almost always*), with higher scores indicating higher stress. Their scores ranged from 29 to 116 points. Of the 29 items, 18 were concerned with psychological stress responses requiring responses on the following five dimensions: liveliness (three items, e.g., “I have been very active”), irritability (three items; e.g., “I have been felt irritable”), fatigue (three items; e.g., “I have been extremely tired”), anxiety (three items; e.g., “I have felt worried or insecure”), and depression (six items; e.g., “I have been depressed”). Physical stress response was assessed using 11 questions on physical complaints (e.g., “I have felt dizzy”). The total psychological and physical stress responses score exhibited high internal consistency (Cronbach's *α* = 0.90).^24^ In the statistical analysis, the stress response score was divided into quartiles, and the group with the highest score was defined as high‐stress teachers, according to the classification protocol in a previous study.^9^, ^21^, ^23^

To assess a participant's job satisfaction, we also used the question in the BJSQ: “My job is rewarding.” Items were rated on a four‐point Likert‐like scale.

We collected data on working hours per day for the previous month with seven response options: (1) <8 h, (2) 8–9 h, (3) 9–10 h, (4) 10–11 h, (5) 11–12 h, (6) 12–13 h, and (7) 13 h or more.

The items regarding the main stressors of teachers were selected based on the opinions of psychiatrists who work at hospitals affiliated with the Public‐School Teachers' Mutual Aid Association and regularly see teachers with mental health problems. Participants were asked to select up to two of the following 14 options as their main stressors: (1) responsibility for student learning; (2) leading extracurricular club activities; (3) school management duties; (4) giving a demonstration lesson; (5) dealing with difficult‐to‐handle students; (6) giving instruction to students (instruction on matters other than learning, including guidance on attitudes toward life and social skills, and brief consultation with the student in difficulty); (7) dealing with overly demanding parents; (8) workload of clerical tasks; (9) relationships with colleagues; (10) relationships with supervisors; (11) unfamiliar work environment due to a transfer; (12) long commuting time; (13) family and private issues; (14) other problems; and (15) nothing in particular. In this study, we compared the main stressors of teachers according to their school type.

### Statistical analysis

Analysis of variance was used to compare the means, and the *χ* ^2^ test was used to compare the proportions. The partial *η* ^2^ was used as the effect size of the analysis of variance. The 0.01 ≥ *η* ^2^ indicates a small effect. The *φ* and Cramér's *V* were calculated for the effect sizes when *χ* ^2^ tests were performed. Values of <0.1 for *φ* and Cramér's *V* were considered negligible. If there was statistical significance and the effect size was more significant than 0.1, multiple comparisons were performed using *z* test with Bonferroni correction. Multiple logistic regression analysis, including adjustment for confounders in a stepwise manner, was conducted to determine the relationship between stressors and teachers in special needs schools. IBM SPSS Statistics for Windows Version 23 (IBM Corp.) was used for statistical analysis, and a significance level of <0.05 (two‐tailed test) was considered statistically significant.

### Ethical considerations

The Ethics Committee of the Kinki Central Hospital of the Mutual Aid Association of Public School Teachers reviewed and approved the Internet‐based survey's research aims, design, and procedures (approval No. 473). This study used existing data. The data were completely anonymous and untraceable and never used to identify personal information. Additionally, the participants used their discretion and willingly responded to the questions, which did not cause psychological distress. Therefore, the ethics committee concluded that informed consent was not required.

## RESULTS

The results of the comparison of the demographic variables are presented in Table 1. Statistical significance was found in age (*F* = 1380.0, *p* < 0.001, partial *η* ^2^ = 0.02), sex (*χ* ^2^ = 11,573.1, *p* < 0.001, *φ* = 0.239), the number of years of continuous employment at the current school (*F* = 289.3, *p* < 0.001, partial *η* ^2^ = 0.004), size of the school (the number of teachers and staff) (*χ* ^2^ = 161,381.6, *p* < 0.001, *φ* = 0.514), working hours per day (*χ* ^2^ = 12,558.5, *p* < 0.001, *φ* = 0.143), job satisfaction (*χ* ^2^ = 1965.7, *p* < 0.001, *φ* = 0.06), and the percentage of high‐stress teachers (*χ* ^2^ = 173.1, *p* < 0.001, *φ* = ‐0.029). Of these variables, the effect sizes of sex, school size, and working hours per day were above 0.1, indicating that special needs schools have more female teachers, a larger school size, and relatively few teachers who work long hours. The partial *η* ^2^ of age was also above 0.01, indicating a small effect size for difference.

**Table 1:** Characteristics of study participants.

	Special needs school		Elementary school		Junior high school		High school		*F*/*χ* ^2^	*p*	Partial *η* ^2^/Cramér's *V*
Age (years), mean (SD)	44.1	(10.94)	41.6	(12.19)	42.0	(11.93)	46.0	(10.98)	1380.02	<0.001***	0.020
Sex, *n* (%)											
Male	5832	(38.5%)	36,546	(38.1%)	31,519	(57.6%)	25,060	(66.7%)	11,573.12	<0.001***	0.239
Female	9327	(61.5%)	59,445	(61.9%)	23,165	(42.4%)	12,539	(33.3%)			
Number or years worked in same school (years), mean (SD)	14.5	(11.60)	14.6	(12.26)	15.0	(12.27)	16.7	(11.78)	289.34	<0.001***	0.004
Size of school workplace (number of teachers and staff), *n* (%)											
1–24	387	(2.6%)	32,529	(33.9%)	13,650	(25.0%)	1089	(2.9%)	161,381.64	<0.001***	0.514
25–49	1054	(7.0%)	55,019	(57.3%)	34,397	(62.9%)	5967	(15.9%)			
50–99	5316	(35.1%)	8223	(8.6%)	6497	(11.9%)	27,931	(74.3%)			
100 or more	8402	(55.4%)	220	(0.2%)	140	(0.3%)	2612	(6.9%)			
Working hours, per day, *n* (%)											
<8 h	1041	(6.9%)	4204	(4.4%)	2185	(4.0%)	1934	(5.1%)	12,558.47	<0.001***	0.143
8 to <9 h	3865	(25.5%)	10,098	(10.5%)	4243	(7.8%)	6359	(16.9%)			
9 to <10 h	5144	(33.9%)	22,440	(23.4%)	9940	(18.2%)	9401	(25.0%)			
10 to <11 h	2713	(17.9%)	21,125	(22.0%)	9919	(18.1%)	7339	(19.5%)			
11 to <12 h	1648	(10.9%)	21,982	(22.9%)	12,649	(23.1%)	7116	(18.9%)			
12 to <13 h	493	(3.3%)	11,130	(11.6%)	9298	(17.0%)	3591	(9.6%)			
13 h and more	255	(1.7%)	5012	(5.2%)	6450	(11.8%)	1859	(4.9%)			
High‐stress group, *n* (%)											
High‐stress group	3686	(24.3%)	22,160	(23.1%)	14,272	(26.1%)	9046	(24.1%)	173.07	<0.001***	0.029
Not applicable	11,473	(75.7%)	73,831	(76.9%)	40,412	(73.9%)	28,553	(75.9%)			
Job satisfaction (“My job is rewarding”), *n* (%)											
Strongly agree	5119	(33.8%)	38,784	(40.4%)	21,090	(38.6%)	11,264	(30.0%)	1965.66	<0.001***	0.057
Agree	8606	(56.8%)	49,799	(51.9%)	28,295	(51.7%)	21,339	(56.8%)			
Disagree	1107	(7.3%)	5977	(6.2%)	4143	(7.6%)	3848	(10.2%)			
Strongly disagree	327	(2.2%)	1431	(1.5%)	1156	(2.1%)	1148	(3.1%)			

*** *p* < 0.001.

We then compared the main stressors among high‐stress teachers according to the type of school and present the results in Table 2. Consequently, the results showed significant differences with effect sizes of 0.1 or higher in the following areas: “leading extracurricular club activities” (*p* < 0.001, *φ* = 0.325), “giving a demonstration lesson” (*p* < 0.001, *φ* = 0.101), “dealing with difficult‐to‐handle students” (*p* < 0.001, *φ* = 0.163), “dealing with overly demanding parents” (*p* < 0.001, *φ* = 0.127), and “relationships with colleagues” (*p* < 0.001, *φ* = 0.103).

**Table 2:** Comparison of main stressors of teachers in special needs, elementary, junior high, and high schools.

Main stressor		Special needs school		Elementary school		Junior high school		High school				
		*n*	(%)	*n*	(%)	*n*	(%)	*n*	(%)	*χ* ^2^	*p*	Cramér's *V*
Responsibility for student learning	Yes	434	11.8%	3107	14.0%	1379	9.7%	1197	13.2%	158.63	<0.001***	0.057
	No	3252	88.2%	19,053	86.0%	12,893	90.3%	7849	86.8%			
**Leading extracurricular club activities**	**Yes**	**12**	**0.3%**	**219**	**1.0%**	**3180**	**22.3%**	**1474**	**16.3%**	**5202.36**	**<0.001** ***	**0.325**
	**No**	**3674**	**99.7%**	**21,941**	**99.0%**	**11,092**	**77.7%**	**7572**	**83.7%**			
School management duties	Yes	904	24.5%	4136	18.7%	3181	22.3%	2421	26.8%	277.32	<0.001***	0.075
	No	2782	75.5%	18,024	81.3%	11,091	77.7%	6625	73.2%			
**Giving a demonstration lesson**	**Yes**	**246**	**6.7%**	**2151**	**9.7%**	**855**	**6.0%**	**260**	**2.9%**	**497.87**	**<0.001** ***	**0.101**
	**No**	**3440**	**93.3%**	**20,009**	**90.3%**	**13,417**	**94.0%**	**8786**	**97.1%**			
**Dealing with difficult‐to‐handle students**	**Yes**	**637**	**17.3%**	**7566**	**34.1%**	**3045**	**21.3%**	**1698**	**18.8%**	**1302.83**	**<0.001** ***	**0.163**
	**No**	**3049**	**82.7%**	**14,594**	**65.9%**	**11,227**	**78.7%**	**7348**	**81.2%**			
Giving instruction to students	Yes	76	2.1%	1084	4.9%	725	5.1%	429	4.7%	63.64	<0.001***	0.036
	No	3610	97.9%	21,076	95.1%	13,547	94.9%	8617	95.3%			
**Dealing with overly demanding parents**	**Yes**	**402**	**10.9%**	**4166**	**18.8%**	**1922**	**13.5%**	**637**	**7.0%**	**787.17**	**<0.001** ***	**0.127**
	**No**	**3284**	**89.1%**	**17,994**	**81.2%**	**12,350**	**86.5%**	**8409**	**93.0%**			
Workload of clerical tasks	Yes	864	23.4%	5351	24.1%	3640	25.5%	2518	27.8%	53.14	<0.001***	0.033
	No	2822	76.6%	16,809	75.9%	10,632	74.5%	6528	72.2%			
**Relationships with colleagues**	**Yes**	**1120**	**30.4%**	**3354**	**15.1%**	**2514**	**17.6%**	**1728**	**19.1%**	**518.83**	**<0.001** ***	**0.103**
	**No**	**2566**	**69.6%**	**18,806**	**84.9%**	**11,758**	**82.4%**	**7318**	**80.9%**			
Relationships with supervisors	Yes	405	11.0%	2466	11.1%	1556	10.9%	741	8.2%	63.93	<0.001***	0.036
	No	3281	89.0%	19,694	88.9%	12,716	89.1%	8305	91.8%			
Unfamiliar work environment due to a transfer	Yes	364	9.9%	1613	7.3%	962	6.7%	659	7.3%	42.62	<0.001***	0.029
	No	3322	90.1%	20,547	92.7%	13,310	93.3%	8387	92.7%			
Long commuting time	Yes	273	7.4%	842	3.8%	683	4.8%	706	7.8%	257.89	<0.001***	0.072
	No	3413	92.6%	21,318	96.2%	13,589	95.2%	8340	92.2%			
Family and private issues	Yes	573	15.5%	2763	12.5%	1542	10.8%	1154	12.8%	68.02	<0.001***	0.037
	No	3113	84.5%	19,397	87.5%	12,730	89.2%	7892	87.2%			
Other problems	Yes	233	6.3%	1122	5.1%	844	5.9%	625	6.9%	44.68	<0.001***	0.030
	No	3453	93.7%	21,038	94.9%	13,428	94.1%	8421	93.1%			
Nothing in particular	Yes	153	4.2%	837	3.8%	505	3.5%	319	3.5%	4.28	0.233	
	No	3533	95.8%	21,323	96.2%	13,767	96.5%	8727	96.5%			

*** *p* < 0.001.

Values in bold indicate the absolute value of Cramér's *V* is more than 0.1.

Post‐hoc pairwise comparisons with Bonferroni correction for the *p*‐value showed statistically significant differences across school types in the frequency of “leading extracurricular club activities,” “giving a demonstration lesson,” “dealing with overly demanding parents,” and “relationships with colleagues.” The stress of “dealing with difficult‐to‐handle students” was significantly more frequent in elementary and middle school teachers than in high school and special needs school teachers. There was also a statistically significant difference between elementary and junior high school teachers.

Extracurricular club activities are usually not organized in special needs or elementary schools. “Giving a demonstration lesson,” “dealing with overly demanding parents,” and “ dealing with difficult‐to‐handle students” were elementary school teachers' most frequently indicated stressors. Among the stressors that were statistically significant and had an effect size of 0.1 or greater, only the “relationships with colleagues” was the most frequently mentioned stressor by teachers in special needs schools. Since this study aimed to elucidate the stressors among teachers in special needs schools, the following multivariate analyses focused on the “relationships with colleagues.”

Multiple logistic regression analysis was performed to examine how frequently teachers in special needs schools experienced stress in relationships with their colleagues compared to teachers in other schools after adjusting for associated factors. The dependent variable was whether or not stress was due to interpersonal relationships with colleagues. The independent variables were age, sex, teaching experience, number of teachers and staff, working hours, job satisfaction, and type of school. Among high‐stress teachers, these variables were used to examine the relationship between stress due to the relationships with colleagues and the type of school in which they work. The results of the multiple logistic regression analysis are shown in Table 3. This aimed to examine the relationship between the type of school and stressors of “relationships with colleagues.” Among high‐stress teachers, the following factors were associated with stressors of “relationships with colleagues”: age (odds ratio [OR] = 1.02 with 95% confidence interval [CI] = 1.02–1.02, *p* < 0.001); being female (OR = 1.26, 95% CI = 1.20–1.32, *p* < 0.001); teaching experience (OR = 0.99, 95% CI = 0.99–1.00, *p* < 0.001); the number of teachers and staff in school being 25–49 (OR = 1.18, 95% CI = 1.04–1.34, *p* = 0.01) or 100 and more (OR = 1.15, 95% CI = 1.07–1.23, *p* < 0.001); job satisfaction (“my job is rewarding”) with response of *agree* (OR = 0.91, 95% CI = 0.86–0.97, *p* = 0.003), *disagree* (OR = 1.12, 95% CI = 1.03–1.20, *p* = 0.004), or *strongly disagree* (OR = 1.45, 95% CI = 1.32–1.60, *p* < 0.001); and being teachers in a special needs school (OR = 1.82, 95% CI = 1.65–2.01, *p* < 0.001).

**Table 3:** Relationship between stress due to interpersonal relationships with colleagues and the other factors among high‐stress teachers.

	Odds ratio	(95% CI)			Wald	*p*
Age (years)	1.02	1.02	–	1.02	115.69	<0.001***
Sex						
Male	Reference					
Female	1.26	1.20	–	1.32	86.61	<0.001***
Number or years worked in same school (years)	0.99	0.99	–	1.00	21.52	<0.001
Number of teachers and staff at school						
1–24	Reference					
25–49	1.18	1.04	–	1.34	6.59	0.010*
50–99	1.04	0.98	–	1.11	1.77	0.184
100 and more	1.15	1.07	–	1.23	13.98	<0.001***
Working hours, per day						
<8 h	Reference					
8 to <9 h	1.15	1.00	–	1.33	3.77	0.052
9 to <10 h	1.01	0.88	–	1.16	0.02	0.892
10 to <11 h	0.92	0.80	–	1.06	1.39	0.238
11 to <12 h	0.91	0.80	–	1.04	1.78	0.182
12 to <13 h	0.95	0.83	–	1.10	0.42	0.515
13 h and more	1.01	0.88	–	1.17	0.04	0.846
Job satisfaction (“My job is rewarding”)						
Strongly agree	Reference					
Agree	0.91	0.86	–	0.97	8.95	0.003**
Disagree	1.12	1.03	–	1.20	8.15	0.004**
Strongly disagree	1.45	1.32	–	1.60	54.78	<0.001***
Type of school						
The others	Reference					
Special needs school	1.82	1.65	–	2.01	137.31	<0.001***

*Note*: Stress due to interpersonal relationships with colleagues was used as the dependent variable in the logistic regression analysis.

* *p* < 0.05

** *p* < 0.01

*** *p* < 0.001.

## DISCUSSION

This study aimed to reveal psychological stressors among teachers in special needs schools. The results of this study showed that (1) teachers in special needs schools do not work as long each week as teachers in other types of schools. (2) Although teachers in special needs schools do not work long hours, the percentage of high‐stress teachers was approximately the same as that of teachers in other types of schools. Furthermore, (3) one of the primary stressors for teachers in special needs schools was “relationships with colleagues.” Even after statistically adjusting for working hours and job satisfaction, it was found that teachers in special needs schools were 1.82 times more likely to experience difficulty in interpersonal relationships with colleagues than teachers in other types of schools.

The results of this study suggest that it is essential for special needs teachers to have favorable relationships with their colleagues. The services and working environments of teachers in special needs schools differ from those in general schools. Most special needs schools in Japan are elementary, junior high, and high schools combined into a single school. For students with intellectual and limb disabilities, who account for the majority of students in special needs schools, the educational content varies greatly depending on the type and degree of the student's disability. Consequently, teachers in special needs schools must develop individualized educational support plans and provide education to achieve independence and social participation per each student's abilities. Moreover, in contrast to general schools, several teachers are assigned to each student, and there are many teachers and office staff in a single special needs school. In addition to teachers, medical professionals are also employed as faculty members. Professionals from various specialties educate students who require highly individualized care. There is no one and only correct way to serve the students with disabilities and their parents. Since teachers' policies, knowledge, and expertise vary, teaching without interprofessional communication can cause conflict when educating students.

When opinions and policies differ from teachers with whom they are expected to collaborate to educate students and deal with parents, colleagues must work together without understanding each other. This often results in psychological stress for teachers. Inoue and Kaneko surveyed 317 special needs school teachers and 202 welfare facility employees concerning their problems and needs.^25^ The results showed that “difficulty in sharing information within the workplace and cooperation with other organizations” was one of the main difficulties. It also pointed to the importance of establishing a cooperation system within the workplace and with other organizations.^25^ The results of their study are consistent with ours.

This study showed that relationships with colleagues become more stressed when job satisfaction is low. Given that job satisfaction may be a confounding factor in the relationship between special needs school teachers and relationship stress, it was necessary to adjust for it statistically. Therefore, the logistic regression model entered job satisfaction as an independent variable. However, even after statistical adjustment for the effect of job satisfaction, teachers in special needs schools were still statistically associated with stress related to relationships with colleagues.

### Limitations

This study is a cross‐sectional survey, and it is not possible to refer to causal relationships. Further longitudinal and prospective surveys and interventions are required. However, this study is worth considering because it is a nationwide survey, and the generalizability of the results obtained is extremely high, reflecting the working environment conditions for teachers of special needs schools in Japan.

## CONCLUSION

This study examined stress factors among public school teachers and found that relationships with colleagues significantly contributed to stress of teachers in special needs schools after statistical adjustment for age, gender, working hours, and job satisfaction. High‐stress teachers in special needs schools tend to experience difficulties in their relationships with colleagues, which may contribute to a high sick leave rate. Today, political efforts are being made in Japan to reduce working hours by reforming the labor style of teachers. However, for teachers in special needs schools, attempts to facilitate interpersonal relationships may be more necessary than reducing overtime working hours.

## AUTHOR CONTRIBUTIONS

Masateru Matsushita and Schuhei Yamamura conceptualized this study. Masateru Matsushita analyzed the data and wrote the manuscript. Schuhei Yamamura supervised the study. Both authors contributed to the manuscript revision and approved the submitted version.

## CONFLICT OF INTEREST STATEMENT

The authors declare no conflict of interest.

## ETHICS APPROVAL STATEMENT

The studies involving human participants were reviewed and approved by the Ethics Committee of the Kinki Central Hospital of the Mutual Aid Association of Public School Teachers (Approval No. 473).

## PATIENT CONSENT STATEMENT

Written informed consent for participation was not required for this study in accordance with the National Legislation and the Institutional Requirements.

## CLINICAL TRIAL REGISTRATION

N/A.

## Data Availability

The datasets presented in this article are not readily available because we cannot publicly provide individual data due to a data provider's regulations. Researchers who are eligible may request access to minimal data sets by contacting the corresponding author on a reasonable request. Requests to access the datasets should be directed to yamamura_s@kich.itami.hyogo.jp.
